# Housing Insecurity and Perceived Mental Health Challenges Among Individuals Living with HIV: Qualitative Insights from Kisumu, Kenya

**DOI:** 10.3390/ijerph23050576

**Published:** 2026-04-29

**Authors:** Patrick Mbullo Owuor, Doreen Obondo, Wicklife Orero, Silvia Odhiambo, Eyram Agbe, Godfred Boateng, Elizabeth Onyango

**Affiliations:** 1 Department of Anthropology, Wayne State University, Detroit, MI 48202, USA; 2 Pamoja Community-Based Organization, Kisumu 40100, Kenya; d.obondo@gmail.com (D.O.); wicklife@pamojapamoja.org (W.O.); 3 School of Public Health, University of Alberta, Edmonton, AB T6G 2R3, Canada; saodhiam@ualberta.ca; 4 Dahdaleh Institute for Global Health Research, York University, North York, ON M3J 1P3, Canada; eyramagbe@cmail.carleton.ca (E.A.); gboaten@yorku.ca (G.B.); 5 Department of Geography and Environmental Studies, Carleton University, Ottawa, ON K1S 5B6, Canada

**Keywords:** housing insecurity, HIV, mental health, stigma, social determinants of health, Kenya

## Abstract

**Highlights:**

**Public health relevance—How does this work relate to a public health issue?**

- Housing insecurity is a public health issue that complicates HIV care and treatment.
- Housing insecurity worsens mental health issues in people living with HIV.

**Public health significance—Why is this work of significance to public health?**

- Housing insecurity is a key social determinant of health and plays a vital role in advancing HIV care and treatment.
- Housing insecurity will be critical for achieving Sustainable Development Goal 3, Good Health and Well-Being.

**Public health implications—What are the key implications or messages for practitioners, policy makers, and/or researchers in public health?**

- Housing in HIV care programs offers valuable insights for HIV prevention and management.
- There is a need to incorporate housing stability and mental health support into HIV programs.

**Abstract:**

Introduction: HIV remains a public health concern despite several decades of effort. In sub-Saharan Africa, where political, environmental, and economic challenges persist, progress toward “Getting to Zero,” including zero new infections and zero HIV-related deaths, has been significantly slow. Although sub-Saharan Africa has seen the successful implementation of HIV/AIDS interventions across behavioral, biomedical, and structural approaches, there has been limited focus on housing insecurity—the inability to access safe, affordable, and stable housing—and mental health among people living with HIV, despite the critical role of housing insecurity in overall health and well-being. Therefore, this study explores how housing insecurity shapes mental health experiences among PLHIV in Kisumu. Methods: Using a qualitative approach, we purposively recruited 70 individuals from households participating in the Pamoja community-based organization’s Orphans and Vulnerable Children (OVC) project. We then conducted in-depth interviews (IDIs) with 30 participants and 4 focus group discussions (FGDs) with 40 adult participants living with HIV (ALHIV). Audio-recorded interviews were transcribed verbatim, translated from Luo into English, and uploaded to Atlas.TI v.23, a qualitative data analysis software. We then performed thematic analysis guided by grounded theory. Results: Our findings showed that housing insecurity was a significant issue for individuals living with HIV. The majority of participants experienced heightened feelings of worry, shame, fear, anxiety, stress, and depression, which negatively impacted their adherence to HIV treatment and care. While some participants showed resilience through acceptance and disclosure, limited resources and ongoing insecurity heightened vulnerability to mental health issues. Discussions: These findings underscore the importance of housing in HIV care programs and offer valuable insights for practitioners and policymakers. The findings highlight the need to incorporate housing stability and mental health support into HIV programs.

## 1. Introduction

The World Health Organization identifies housing as a key structural factor influencing health outcomes, particularly in vulnerable populations [1]. Globally, over 1.6 billion people live in inadequate housing, and approximately 100 million are homeless [2]. Housing insecurity is not only about shelter but also encompasses affordability, quality, safety, and security of tenure [3]. These housing deficits have been linked to poor mental health outcomes, including anxiety, depression, and psychosocial stress [4]. For people living with HIV (PLHIV), stable housing is associated with improved antiretroviral therapy (ART) adherence, reduced mortality, and better psychological wellbeing [5,6]. Despite numerous efforts to address HIV, sub-Saharan Africa continues to lag in treatment outcomes. Although structural barriers to prevention and treatment have been extensively studied [7,8,9], ongoing vulnerabilities caused by increasing resource insecurities remain a significant threat. The implementation of sustainable goals—SDGs 1, 2, 3, and 6—has increased access to food, reduced poverty, improved healthcare access, and lessened water insecurity [10]. As a result, these interventions have helped decrease vulnerabilities and risks of HIV. While housing insecurity—defined as the lack of safe, stable, affordable, and adequate housing—has emerged as a critical social determinant of health globally [11], its relevance in HIV/AIDS in sub-Saharan Africa remains underexplored.

Sub-Saharan Africa bears two-thirds of the global HIV burden [12], yet research on housing insecurity and mental health among PLHIV is sparse. Previous studies have mainly explored the link between housing insecurity and homelessness in relation to HIV incidence and transmission, with evidence indicating that poor housing conditions increase vulnerability to HIV through higher risk behaviors, difficulties in treatment adherence, and obstacles to accessing care [13]. While these studies have largely centred on the biomedical and epidemiological aspects of HIV, there has been less emphasis on the psychosocial effects of housing insecurity. Moreover, despite evidence that stress, anxiety, and depression can negatively impact treatment adherence and overall well-being [14], the mental health issues faced by people living with HIV in unstable housing conditions are still not well understood. While rapid urbanization and economic inequality further worsen housing insecurity in the sub-Saharan Africa region [15], regional policies have not incorporated housing support into HIV care frameworks. Additionally, despite high HIV prevalence and widespread housing issues, the connection between housing conditions and mental health among PLHIV remains underexplored in the region.

In Kenya, an estimated 1.4 million people live with HIV, with prevalence concentrated in western counties such as Kisumu, Siaya, and Homa Bay [16]. Kisumu county, for example, has more than triple the national average of 3.7% [17,18]. The county also faces severe housing deficits, with many residents living in informal or semi-permanent structures that lack adequate ventilation, weatherproofing, and security [19]. While national HIV programs have integrated nutrition, economic strengthening, and stigma reduction, housing support remains largely absent from care frameworks. These structural vulnerabilities are intensified by poverty, rural–urban migration, and limited infrastructure in peri-urban settlements and rural areas.

Despite these realities, few empirical studies in Kenya have examined how housing insecurity specifically affects mental health among PLHIV, especially through the lens of their lived experiences. Therefore, this study explores how housing insecurity shapes mental health experiences among PLHIV in Kisumu. This research contributes to the evidence base on social and structural determinants of HIV outcomes, offering insights for integrated housing and mental health interventions within HIV care programs.

### Theoretical Framework

This study employs the syndemic approach, which helps explain how social and structural determinants may intensify interactions among two or more health issues [14]. Singer popularized the term in 2009 to describe how social and lifestyle behaviors—such as substance abuse and violence—are associated with HIV/AIDS and other behavioral problems. In his analysis, Singer emphasized the importance of examining the co-occurrence of multiple health conditions and the social, political, and environmental factors that drive them [20]. In HIV, the syndemic approach has been instrumental in highlighting how comorbidities are exacerbated by intersecting factors such as poverty and stigma, and vice versa [21,22], leading to life-changing interventions in the management of HIV/AIDS. For example, studies on social stigma have contributed to increased adherence to HIV treatment, while socioeconomic interventions have improved treatment outcomes by increasing access to essential services such as food, leading to reductions in viral load and increases in CD4 counts [23,24,25]. Moreover, the persistent focus on other infectious diseases, such as tuberculosis, sexually transmitted infections, such as syphilis, and vector-borne diseases, such as malaria, has significantly contributed to slowing disease progression, reducing new infections, and curbing HIV-related deaths [26,27]. Recently, the focus of the syndemics framework in HIV and non-communicable diseases has widened to include mental health. While this focus on mental health is not new, the social and structural factors have largely centered on socioeconomic status and indicators such as food and water security [26,28]. Housing insecurity and HIV, especially in low and middle-income countries, have received minimal attention.

## 2. Methods

### 2.1. Study Setting

This study was conducted in Kisumu West and Seme sub-counties of Kisumu County, Kenya, which are predominantly rural areas with pockets of peri-urban settlements near Kisumu City. The local economy is driven by rain-fed subsistence farming, fishing along Lake Victoria, and informal transport services, such as motorcycle taxi (*boda-boda*) [29]. Housing structures range from mud-walled huts with thatched roofs to semi-permanent and iron-sheet houses, many of which lack secure doors, windows, or proper flooring. Additionally, Kisumu County has one of the highest HIV prevalence rates in Kenya [16,30].

### 2.2. Research Design

We employed a qualitative descriptive design to capture first-hand accounts of housing and mental health experiences among PLHIV [31]. This design was chosen to enable a nuanced understanding of participants’ perspectives without imposing pre-existing theoretical frameworks [32].

### 2.3. Study Population and Recruitment

#### 2.3.1. About Pamoja CBO

Pamoja Community-Based Organisation (CBO) is a grassroots organisation established in 2007 to empower communities to identify and address the most significant needs affecting their well-being. Registered with the Ministry of Labour and Social Protection, the organisation implements programmes that address the immediate needs of vulnerable communities while building longer-term resilience. The organisation focuses on community health and programming; water and sanitation; education and training; and food security and the environment. The Orphans and Vulnerable Children (OVC) Project is one of the interventions that the organisation implemented between 2013 and 2025, towards building adaptive strategies of families and children affected by HIV and AIDS so that they can meet their health, economic, education, and social development needs.

#### 2.3.2. Sampling, Inclusion, and Exclusion Criteria

Participants were purposively sampled from 70 individuals in households enrolled in the Orphans and Vulnerable Children (OVC) Project implemented by Pamoja Community-Based Organization (CBO) between 2013 and 2023. Inclusion criteria were: (a) being HIV-positive and aged 18 years or older; (b) residing in Kisumu West or Seme; and (c) willingness to discuss personal housing and mental health experiences. To ensure sufficient sample size for saturation, we relied on Bernard Russell’s recommendation of 30 individuals [33]. Thus, the final sample included in-depth interviews (IDIs) with 30 participants, evenly split across sub-counties, with 15 from each sub-county. It also included four focus group discussions (FGDs) with 40 participants—19 from Seme (9 women, 10 men) and 21 from Kisumu West (12 women, 9 men). Each FGD session consisted of 9–12 participants, with recruitment focused on achieving diversity across gender, age, and marital status. To introduce variation in housing, recruitment also targeted different housing materials and ownership types, including rented and owned properties.

### 2.4. Data Collection

Data were collected between July and August 2023 using semi-structured interview guides that explored housing conditions and perceived adequacy; experiences of safety and insecurity; mental health symptoms and coping strategies; and experiences of HIV-related stigma.

Research Instruments: The initial guides for in-depth and focus-group discussions were developed from existing literature on HIV, housing insecurity, and mental health. To ensure cultural relevance, local elders reviewed the tools and provided feedback on key topics. To improve accessibility and clarity, the instruments were translated from English into two widely spoken regional languages—Dholuo and Swahili—by two authors (W.O. and D.O.), who are native speakers fluent in both languages. To prevent loss of information during translation, the instruments were back-translated into English by the same researchers. One non-native researcher (G.B.) then reviewed the back-translated version against the original to check for consistency [20].

In-depth interviews (IDIs): In-depth interviews were included in this study to capture personal experiences and sensitive issues that would be difficult to discuss in group discussions and to gain insight into individual perceptions [34]. We conducted IDIs with 30 participants (17 women and 13 men), evenly recruited from both Seme and Kisumu West sub-Counties. Interviews were conducted by qualitative researchers in the participants’ homes using their preferred language. Interviews included questions such as: Could you please describe the type of housing most people in your community live in? How do most families afford and maintain the kind of housing? What are your experiences with the physical structure of the house? What do you observe when it rains, is hot, is windy, etc.? Tell me about the air quality in your house. Do you think your housing units affect the treatment you get?

Focus Group Discussions (FGDs): To capture community-wide perceptions on housing insecurity, HIV, and mental health, triangulated in-depth interviews with FGDs [35,36]. We conducted four FGDS with 40 participants, two from each sub-county. Each session comprised 10 people. To encourage discussion and reduce power dynamics and cultural influences, FGDs for both men and women were conducted separately. One research assistant moderated the discussions, and the other took notes to ensure detailed documentation, including nonverbal cues. FGD sessions included questions such as: What is your overall satisfaction with this neighborhood? Can you describe any experiences of violence in this community? How does people’s health change due to the changes in their housing structure? What are some specific fears your family members express? Would you please describe any experience you or a neighbor has had in the past year related to housing challenges?

To minimize distractions and maintain confidentiality, IDIs were conducted in the participants’ homes, while FGDs were held in local nearby schools. All sessions were audio-recorded with consent, lasted on average two hours for FGDs and an hour for the IDIs, and took place in private, neutral locations.

### 2.5. Data Analysis

Two researchers (D.O. and W.O.), fluent in Dholuo and Swahili, transcribed the audio recordings and, where necessary, translated them into English. To assist with coding, the researchers initially created a coding scheme grounded in existing literature on the topic and based on coding the first two IDIs and one FGD. A coding scheme was considered necessary to structure the coding process and avoid over-coding [37]. The coding scheme was then imported into Atlas.TI (a qualitative research analysis tool) to help track the coding process, organize, and facilitate subsequent coding. To ensure intercoder reliability [38,39,40], first, two researchers (D.O. and W.O.) independently coded and back-coded a subset of transcripts. During this initial coding, new codes were allowed and added to the coding scheme as they emerged. This deductive and inductive process allowed researchers to reflect on the codes and make necessary adjustments throughout the coding process [41]. This process also emphasized the researcher’s ongoing, reflexive engagement with the knowledge production process, particularly how one’s theoretical position and unique experiences influence interpretation of the data [42]. In the second round of coding, the research team, comprising researchers familiar with housing insecurity and mental health (P.M.O. and E.O.), met to discuss convergence and divergence in the coding process, during which codes were merged or subdivided [39,40]. Lastly, to validate the new set of codes, one team member (G.B.), who was not involved in the first and second stages of the coding process, evaluated the application of the new codes. All researchers then participated in a brainstorming session to assess the data for patterns and code frequencies. They then grouped related codes into categories based on these emerging patterns, which ultimately led to the identification of themes.

### 2.6. Ethical Considerations

Ethical approval was obtained from the AMREF Health Africa Ethics and Scientific Review Committee (ESRC-P1396-2023). Written informed consent was obtained from all participants. Pseudonyms were used to protect confidentiality, and referrals to psychosocial support services were provided when needed. Participants received KES 500 (US$4) per interview session as reimbursement.

## 3. Results

### 3.1. Sociodemographic Characteristics of Study Participants

The average age of IDI participants was 45 years, with a range from 24 to 65 years. Most women were slightly younger, averaging 43 years, and tended to be less educated than men. A majority (60%) of women had no formal education, while only 30% had completed some primary schooling. Households generally had larger family sizes, with at least five dependent children, and some up to nine. Most women were married (80%), and 17% were widowed. Most had at least three live births, reflecting significant family responsibilities. Farming was the main occupation for most, with 60% citing it as their primary livelihood.

### 3.2. Emerging Themes

Our findings revealed that, although people living with HIV face numerous challenges, housing insecurity remains a major concern for many. Most participants reported increased feelings of worry, shame, fear, anxiety, stress, and depression, which adversely affected their adherence to HIV treatment and care. While some individuals demonstrated resilience through acceptance and disclosure, limited resources and persistent insecurity heightened their susceptibility to mental health problems. We organized these experiences of perceived poor mental health into six thematic areas (Table 1), illustrating how housing insecurity interacted with social and structural factors to further worsen HIV/AIDS.

### 3.3. Stigma and the Emotional Strain of Living in Poor Housing Conditions

Our study participants reported feelings of embarrassment, hopelessness, and diminished self-worth, tied to poor housing. They also reported feelings of emotional distress as they reflected on self-worth, dignity, and respect within the community. As the following participants explained:

“When someone lives in a good house, you have no stress, unlike my house, where I feel embarrassed when I am expecting visitors. When you are a young woman with a bad house structure, people view you as a poor woman because of the house you live in.”

(IDI, Kisumu West, 35-year-old female)

“Housing is very important to me as a woman. I feel embarrassed when people discuss their homes, upcoming improvements, or future purchases. For example, my friend recently bought a high-quality sofa because she has a spacious house with cement floors and a sturdy roof. I can’t afford such things because my home is limited in space, and my floors and walls are in poor condition. This makes these conversations quite stressful, especially when they pop up repeatedly in our small business groups. Sometimes, I just wish I could hide and hope no one asks about my plans.”

(FGD, Kisumu West, 45-year-old female)

Some participants in our study also reported feeling stigmatized, not being respected by community members, and feeling ashamed of the condition of their houses. For these participants, the home was viewed as a symbol of status.

“Last time I went to the chief’s office, it was because my house did not have a sanitation facility. During that time, my family used the surrounding bushes as latrines. The chief scolded me, called me names, and everyone was laughing at me. I felt exposed and vulnerable.”

(IDI, Seme, 40-year-old male)

“Housing is the only way you earn respect. It starts when you know you cannot host important family events because you are worried about the condition of your house, the limited space, and the dusty floors. I remember that at our end-of-year family gatherings, I always had to give an excuse for not being able to host a Christmas lunch or dinner.”

(FGD, Seme, 38-year-old female)

The housing situation also affected when and how the children with HIV slept and took their medication. Some participants reported that their children felt uncomfortable when other family members visited because the small house made it obvious that they were on HIV medication.

“It is a small house, so everything is done in the eyes of everyone. When some family members visited, their children wanted to know why my children were taking drugs every day, yet they are okay and healthy… My children were so frustrated and had difficulty sleeping and staying on medication.”

(IDI, Kisumu West, 42-year-old female)

“For us men, it is easy to hide the stress or concerns we have because we have to remain strong for our families, but, as the other member has said, housing is very critical, especially for me as someone on HIV medication. I need a private room away from my children, so I do not have to wake them up when I’m taking my medications. Sometimes you fall sick and want comfort, but you can’t get it. We still share our bed with a 4-year-old child. So, you see, these are difficult things.”

(FGD, Seme, 48-year-old male)

### 3.4. Psychosocial Consequences

The poor housing conditions were also reported to contribute to a sense of insecurity and generate anxiety among participants. Some participants also expressed constant unease due to community safety threats such as robberies and violence.

“My house isn’t secure; the door and windows are nearly falling apart. At night, around 9:00 PM, the dogs begin barking, making you worry about being attacked by wild animals or robbers. At that time, even if you were to wake up to take your medicine, you won’t. You would rather wait until it is morning and it feels safe.”

(IDI, Seme, 48-year-old female)

“During the last rainy season, I missed several clinic appointments because I was busy with house repairs. I think it was during this time that I developed some of my current medical conditions. I would wake up to repair damaged walls and floors. It was even worse when it rained at night, causing the roof to leak and sometimes keeping me awake.”

(FGD, Kisumu West, 52-year-old male)

Our participants also reported feeling distressed from cooking in cramped, poorly ventilated homes, which they said affected their physical health through suffocation and smoke inhalation. Other participants shared how crowded their small homes made them feel stressed and worried about catching airborne diseases during outbreaks.

“I have a very large family, and this house is small. When everyone is at home, we feel really squeezed. Lack of windows makes it worse. We are always worried when sickness is going around because we are likely to get sick. Inside the house, the air is bad, especially when it is hot outside.”

(IDI, Kisumu West, 53-year-old male)

“Though we are often reminded during clinic visits that HIV management requires good nutrition and a clean environment, poor housing makes it very difficult to meet these expectations. You may have to delay cooking because it is raining, your floors are wet, the roof is leaking, or the house is stuffy with smoke from firewood. I think some of these conditions have contributed to my persistent chest problems.”

(FGD, Seme, 42-year-old male)

### 3.5. Financial Constraints

Participants also shared ongoing concerns about their struggle to repair their dilapidated homes and how they frequently engaged in mental calculations about repair expenses, existing financial pressures, and the costs related to HIV treatment. For example, one participant explained that she constantly balanced the costs of transportation to the clinic, lab requests from other doctors, and the need to repair her house, prioritizing urgent home repairs and delaying lab tests.

“I need to keep my clinic appointments, which has led me to postpone repairs around the house. However, during the last rainy season, my house was damaged, forcing me to make a choice.”

(IDI, Seme, 47-year-old female)

“I recall that my doctor once advised me to undergo some tests due to health issues I was facing. However, since I was also renovating my house at the time, I found it difficult to choose between paying for the lab tests or repairing my home. Given the extensive damage, I decided to prioritize fixing the house, and I have not yet taken the tests.”

(FGD, Kisumu West- 44-year-old male)

Similarly, participants noted challenges in balancing home repairs and purchasing food for their families, which affected the household’s nutritional wellbeing. These issues were more frequent during seasons with crop failures, forcing families to rely on food bought from outside sources.

“Keeping my family’s nutrition on track has been difficult, especially for the little ones relying on fortified flour for meals. Since they recently started medication, I was told to use fortified flour to help increase their weight. However, balancing this with other household food needs makes it challenging.”

(IDI, Kisumu West, 43-year-old female)

“Honestly, there have been many times when my family skipped meals to urgently repair something in the house. Most recently, after a heavy storm, the door fell, and part of the roof broke. The next day, I used our limited funds to buy materials and get help fixing the damage, even though it meant missing meals. Imagine being on ARVs daily with little or no food for four days.”

(FGD, Seme, 38-year-old male)

### 3.6. Sleep Deprivation

Our participants reported that insufficient sleep was a prevalent issue among families living in inadequate housing. This was attributed to factors such as small-sized rooms or damaged roofs and walls. During the rainy season, most families remained awake because their sleeping areas became wet.

“When it rains heavily, we are forced to look for where the roof leaks and cover them… We can’t sleep comfortably because we have to share the dry areas when it is raining. The following day, you feel so heavy-headed, dizzy, and not ready to do anything. For me, once I lose sleep, my memory is interfered with, and I forget even when I need to take medication.”

(IDI, Seme, 49-year-old male)

“I have four children, and since our home only has one bedroom, we all end up sharing the living room. They sleep on mats next to my bed, which means there’s not much privacy. Rainy seasons can be quite stressful because if it rains when no one is home, it can wet our bedding, medication, and food, which is really upsetting.”

(FGD, Kisumu West, 40-year-old female)

### 3.7. Intimate Partner and Domestic Violence

Participants described multiple forms of violence, including sexual violence, attributed to the small room and reduced privacy. Other participants also reported on community-level cases of sexual violence related to poor housing conditions.

“Sexual violence happens mostly in the household because of how small they are. Young girls, mostly teenagers, find themselves in risky situations when they have to share private spaces with their brothers and cousins, which leads to things happening.”

(IDI, Kisumu West, 42-year-old female)

“I regularly attend community chief meetings where domestic cases are discussed. Unfortunately, nearly every month, reports emerge of criminals breaking into homes to commit sexual violence. They take advantage of the poor housing conditions. This is heartbreaking and traumatic, especially for young girls who often suffer, sometimes contracting HIV or becoming pregnant. I hope measures can be implemented to prevent these incidents.”

(FGD, Seme, 55-year-old man)

### 3.8. Social Support

Participants’ narratives revealed that social support played a critical role in mitigating the effects of housing insecurity and sustaining HIV care routines, yet access to such support was uneven. Where support was available, it came in the form of financial contributions from relatives, such as siblings or spouses, which helped meet basic needs like food and savings.

“We have received support from our relatives and friends to improve the structure of this house. It has taken us a long time to make these improvements. I split the money I get and use some to feed the family and save the rest so we can make changes in the house.”

(IDI, Seme, 47-year-old male)

“I am a member of an informal group that meets monthly and contributes money to help each other. Last time I got my share, I made improvements on my floor, which was always dusty. Right now, coughs have decreased, and my children no longer complain of rashes.”

(FGD, Kisumu West, 33-year-old female)

Community-based organizations and aid initiatives—such as Pamoja CBO—were instrumental in improving housing conditions by providing building materials, cash transfers, or complete home construction. These interventions not only addressed structural inadequacies but also enhanced psychosocial well-being by reducing stress linked to poor living environments:

“If not for Pamoja CBO, I doubt we would be a family today. They supported many families here by providing nails, iron sheets, a door, and building houses for widows and vulnerable families. At least for those who are on medication, you have one thing taken care of an you can have peace of mind.”

(IDI, Kisumu West, 52-year-old female)

“My friends’ support arrived at a critical moment when my husband and I were close to separation. Our house was in poor condition, and I worried about our health and that of our children. We kept falling ill so often that healthcare providers became concerned. Although I didn’t tell them about the house, I suspect it was a factor in our frequent illnesses.”

(FGD, Kisumu West, a 46-year-old female)

However, some participants highlighted the absence or insufficiency of social support, which left individuals more vulnerable to the compounded effects of poverty, stigma, and housing insecurity. Those without supportive relatives or access to aid programs reported having to rely solely on irregular earnings or casual labor, making both housing improvement and consistent HIV care more challenging.

“We do not participate in the social support groups here due to stigma. As a result, we have also lost support from people who attend these groups. Members in these groups receive money through their savings, which they can use to improve their homes and nutrition.”

(IDI, Seme, 45-year-old Male)

“My friends have a group where they can take out loans from a microfinance institution and support each other during tough times. However, I can’t join because of how they speak about people with HIV. I need peace of mind, even if it means missing out on those opportunities. Currently, I do odd jobs and save money. My partner also contributes whenever she earns. I’m confident we’ll gather enough funds to improve our house. At the moment, health is our top priority, and with rising costs for medication and food, the house can wait.”

(FGD, Seme, 54-year-old male)

## 4. Discussion

This study sought to explore the perceived mental health impacts of housing insecurity among people living with HIV in Kisumu, Kenya. Our research identifies six key themes where housing insecurity adversely affects the psychosocial health of HIV-positive individuals: (a) Stigma, (b) psychosocial consequences, (c) financial constraints, (d) sleep deprivation, (e) intimate partner and domestic violence, and (f) social support.

Housing insecurity was found to be both stigmatizing and a facilitator of stigma among individuals living with HIV. Our study found that housing insecurity among people living with HIV was a source of stigma. For many individuals, poor housing conditions lead to shameful encounters with people in their social networks. These circumstances made them feel stigmatized, less worthy, and of low social status, and hence, they refrained from participating in community social activities. While stigma in HIV has been widely documented, stigma due to housing conditions has not been examined. Our study reveals how poor housing is stigmatizing and perpetuates conditions of stigma for people living with HIV. While our findings resonate with studies in Zambia and Ethiopia, where poor housing was both a source and symbol of social marginalization for PLHIV [43], these findings are the first to suggest housing conditions as a facilitator of HIV stigma. Additionally, in HIV studies, social stigma has been the central focus with emphasis on social interactions [44]. In this study, we recognize that stigma related to housing security is more deeply rooted in structural issues. This perspective is thus helpful for developing a new way of understanding stigma, one that highlights the importance of addressing broader structural inequalities.

Psychosocial consequences resulted from poor housing conditions such as damaged walls, leaking roofs, and wet floors. In this study, participants’ narratives reflected how poor housing conditions were a source of embarrassment, hopelessness, and diminished self-worth. These findings are consistent with research from South Africa and Uganda, where substandard housing was linked to feelings of shame and social exclusion [45,46]. The psychosocial toll appears particularly acute for women, whose social standing is often tied to household appearance—a pattern consistent with gendered analyses of material deprivation [47]. Furthermore, persistent fears of theft, violence, and unsafe surroundings align with the ecological model of health, which recognizes the environment as a determinant of well-being [48]. Similar associations between neighborhood safety concerns and chronic stress among PLHIV have been reported in informal settlements in South Africa [49] as well as in other locations around the world [50]. Moreover, housing conditions such as leaking roofs, damaged walls, and exacerbated safety concerns among individuals further complicate psychosocial health. These findings resonate with broader evidence linking housing insecurity to heightened anxiety, depression, and emotional strain across diverse populations [51]. For individuals already coping with the daily demands of HIV care, such persistent stressors may exacerbate vulnerability, erode, and threaten adherence to treatment [52]. Similar findings from Uganda and South Africa show that poor housing quality and overcrowding are linked to psychosocial stress, increased tensions with partners, and higher vulnerability to illness [7,48]. Thus, this study reveals the multifaceted ways in which housing insecurity impacts mental health. It is worth noting that the conditions of the housing structure and the neighborhood where people live are critical to mental health.

Financial constraints due to housing insecurity complicated treatment plans and influenced individual choices. Participants shared struggles with repairing or maintaining housing, threats of eviction, and a lack of safe spaces for cooking or storage. Additionally, we found that people sometimes delay or miss their clinic appointments because they need time for repairs, or they might postpone treatment or paying for laboratory tests due to ongoing housing repair costs. Moreover, we found that individuals rationed their food, bought fewer groceries, or skipped meals for several days to save money for housing repairs. The impact of financial resources on HIV/AIDS is well documented and includes reduced access to services, low uptake of medical care, poor nutrition, mental health issues, and difficulties with treatment adherence, leading to poorer treatment outcomes [53,54,55,56,57]. Despite this elaborate focus on the impact of financial constraints on HIV treatment outcomes, less has been documented on the link to housing insecurity, especially among people living with HIV in rural communities. This study highlights significant insights into the impact of housing insecurity on people living with HIV. Enhancing housing stability through subsidies, repairs, or community shelter initiatives can provide valuable psychosocial and biomedical support. Such measures can reduce chronic anxiety and support improved health, better medication adherence, and overall wellbeing.

We also found that poor housing conditions compromised sleep quality. Larger families, for example, were forced to share sleeping areas. Additionally, during rainy seasons, families with damaged roofs, floors, or walls found it difficult to sleep at night. Evidence suggests that poor sleep exacerbates disease progression, opportunistic infections, and AIDS-related deaths [58]. Moreover, studies of sleep among adults with HIV have primarily focused on disease-related and psychosocial health issues [59]. Despite this evidence, in sub-Saharan Africa, the role of housing insecurity in sleep disruption among people living with HIV has received minimal attention. This study extends the literature on sleep and HIV/Aids by providing insights into the role of housing insecurity.

Housing insecurity was also found to exacerbate intimate partner and domestic violence, thereby impacting psychosocial well-being. For example, overcrowded living conditions were described as providing conditions for violence, such as physical and sexual abuse, to occur. Additionally, the lack of adequate sleeping spaces for adults blurs privacy, leading to emotional violence. These findings are consistent with evidence from Tanzania and Malawi linking cramped housing to interpersonal conflict and reduced mental health [60]. The constant negotiation of space—especially in multigenerational households—intensified stress and strained family relationships [61].

While many families noted some elements of social support as they navigated housing challenges, for some individuals, housing insecurity eroded their social support. The findings reinforce that social support—both formal and informal—can serve as a critical buffer against the adverse effects of housing insecurity among people living with HIV [44,62]. Consistent with previous studies in sub-Saharan Africa, supportive family members facilitated treatment adherence through reminders, assistance with obtaining ARVs, and emotional reassurance [63]. Disclosure within the household appeared to be a pivotal enabler, reducing secrecy around medication and fostering a supportive environment for ongoing care. Similarly, targeted assistance from community-based organisations such as Pamoja CBO had tangible impacts on housing quality, which in turn may reduce psychosocial stress and enhance health stability [64]. These behaviours align with the stress-buffering hypothesis [61], suggesting that resilience can mitigate the negative mental health impacts of housing insecurity.

Additionally, our findings also reveal stark disparities as some participants lacked access to any meaningful support—whether from family, community networks, or external programmes. The absence of social and material assistance compounded existing vulnerabilities, forcing individuals to rely solely on precarious, low-paying work to meet essential needs. This gap echoes broader critiques of uneven social protection coverage in Kenya, where aid is often fragmented, project-dependent, and fails to reach the most marginalised [65]. For PLHIV, these shortcomings risk perpetuating cycles of housing insecurity, economic hardship, and psychosocial strain, thereby undermining treatment adherence and overall well-being. The co-occurrence of poor housing, unemployment, and HIV illustrates a syndemic pattern [66], where overlapping vulnerabilities intensify mental health risks. Similar patterns have been documented in urban U.S. studies with low-income PLHIV [6], suggesting that the syndemic model has cross-contextual relevance. Addressing these gaps requires multi-level interventions that integrate housing support into HIV care programming, strengthen informal care networks, and expand equitable access to formal social protection schemes.

### 4.1. Policy Implications

Our findings highlight the need to advocate for government and donor funding to support housing initiatives for people living with HIV. Since structural shortcomings cause clearly visible negative mental health effects for PLHIV, it is crucial to go beyond biomedical solutions when designing treatment programs for this group. More consistent funding could enable housing assessments conducted by non-governmental organizations and community groups, such as Slum Dwellers International, which have led remuneration efforts in informal settlements across Africa [67]. Additionally, this study shows that community-led initiatives are key in addressing the complex ways housing insecurity and mental well-being intersect. Therefore, planning and implementing community-led projects can help communities and researchers identify and tackle existing tensions that worsen anxiety and stress, as well as strengthen social ties that may serve as resources for future efforts.

### 4.2. Strengths and Limitations

While rich in qualitative depth, this study was cross-sectional and limited to two sub-counties in Kisumu, and findings may not generalize to all PLHIV in Kenya. Furthermore, since the participants were exclusively chosen from beneficiaries of Pamoja CBO, their experiences might not fully reflect the circumstances of the most vulnerable PLHIV in Kenya, who lack any community support. Additionally, because participants for the study were previous beneficiaries of the organization, their perspectives might be biased, which could also limit the ability to generalize the findings. While this study was conducted in a rural setting and highlighted often-overlooked issues, it primarily focused on structural conditions and maintenance, thus excluding the housing challenges faced by urban dwellers living in rental housing. Future research could be longitudinal and use mixed methods designs to quantify the mental health impacts of housing interventions.

## 5. Conclusions

Housing insecurity—characterized by financial hardship, stigma, diminished social support, and higher physical health risks—can add complexity to HIV/AIDS and mental health issues. Although biomedical treatments for HIV/AIDS are more accessible, individuals living with the condition—especially in resource-limited areas—still face substantial structural obstacles that can impede their treatment success. Improving housing conditions alongside HIV treatment may enhance psychosocial well-being and treatment outcomes. Consequently, incorporating housing interventions into HIV care strategies presents a promising way to alleviate mental health burdens and reduce disparities related to HIV. Therefore, we advocate for including mental health services as part of interventions for PLHIV facing housing inadequacies.

## Figures and Tables

**Table 1:** Emerging themes and their descriptions.

Theme	Description
Stigma and the Emotional Strain	Perceived stigma from housing conditions
Psychosocial Consequences	Perceived mental health challenges including anxiety, stress, worry, and depression
Financial Constraints	Economic and financial challenges related to house maintenance and healthcare
Sleep Deprivation	Lack of sleep due to housing conditions including space and the environment
Intimate Partner and Domestic Violence	Violence within the house including sexual, physical and emotional violence
Social Support	Support received from relatives and friends

## Data Availability

The original contributions presented in this study are included in the article. Further inquiries can be directed to the corresponding authors.
